# Validation of a genetic risk score for Arkansas women of color

**DOI:** 10.1371/journal.pone.0204834

**Published:** 2018-10-03

**Authors:** Athena Starlard-Davenport, Richard Allman, Gillian S. Dite, John L. Hopper, Erika Spaeth Tuff, Stewart Macleod, Susan Kadlubar, Michael Preston, Ronda Henry-Tillman

**Affiliations:** 1 Department of Genetics, Genomics & Informatics, College of Medicine, University of Tennessee Health Science Center, Memphis, Tennessee, United States of America; 2 Genetic Technologies Ltd, Fitzroy, Victoria, Australia; 3 Centre for Epidemiology and Biostatistics, University of Melbourne, Parkville, Victoria, Australia; 4 Phenogen Sciences Inc, Charlotte, North Carolina, United States of America; 5 Genomics Core, Winthrop P. Rockefeller Cancer Institute, University of Arkansas for Medical Sciences, Little Rock, Arkansas, United States of America; 6 Division of Medical Genetics, University of Arkansas for Medical Sciences, Little Rock, Arkansas, United States of America; 7 Center for Diversity Affairs and Inclusion, Winthrop P. Rockefeller Cancer Institute, University of Arkansas for Medical Sciences, Little Rock, Arkansas, United States of America; 8 Department of Surgery, College of Medicine, University of Arkansas for Medical Sciences, Little Rock, Arkansas, United States of America; Ohio State University Wexner Medical Center, UNITED STATES

## Abstract

African American women in the state of Arkansas have high breast cancer mortality rates. Breast cancer risk assessment tools developed for African American underestimate breast cancer risk. Combining African American breast cancer associated single-nucleotide polymorphisms (SNPs) into breast cancer risk algorithms may improve individualized estimates of a woman’s risk of developing breast cancer and enable improved recommendation of screening and chemoprevention for women at high risk. The goal of this study was to confirm with an independent dataset consisting of Arkansas women of color, whether a genetic risk score derived from common breast cancer susceptibility SNPs can be combined with a clinical risk estimate provided by the Breast Cancer Risk Assessment Tool (BCRAT) to produce a more accurate individualized breast cancer risk estimate. A population-based cohort of African American women representative of Arkansas consisted of 319 cases and 559 controls for this study. Five-year and lifetime risks from the BCRAT were measured and combined with a risk score based on 75 independent susceptibility SNPs in African American women. We used the odds ratio (OR) per adjusted standard deviation to evaluate the improvement in risk estimates produced by combining the polygenic risk score (PRS) with 5-year and lifetime risk scores estimated using BCRAT. For 5-year risk OR per standard deviation increased from 1.84 to 2.08 with the addition of the polygenic risk score and from 1.79 to 2.07 for the lifetime risk score. Reclassification analysis indicated that 13% of cases had their 5-year risk increased above the 1.66% guideline threshold (NRI = 0.020 (95% CI -0.040, 0.080)) and 6.3% of cases had their lifetime risk increased above the 20% guideline threshold by the addition of the polygenic risk score (NRI = 0.034 (95% CI 0.000, 0.070)). Our data confirmed that discriminatory accuracy of BCRAT is improved for African American women in Arkansas with the inclusion of specific SNP breast cancer risk alleles.

## Introduction

Racial disparities among women of color is evident. African American women have higher mortality rates, poorer breast cancer survival rates, and are more likely to develop early onset basal-like breast tumors that are non-responsive to hormone receptor therapy compared with European American women [1–3]. In the state of Arkansas breast cancer incidence rates have dramatically decreased due to state-funded breast and cervical cancer screening and diagnostic services for eligible Arkansas women. However, mortality rates due to breast cancer among African American Arkansas women are amongst the highest in the nation [1]. Breast cancer risk assessment tools, such as the BCRAT (also known as the Gail model) were developed by scientists at the National Cancer Institute to estimate a woman’s risk of developing invasive breast cancer within the next 5 years and within her lifetime (up to age 90). The BCRAT considers race/ethnicity, age, breast cancer risk factors that influence estrogen production (such as menopausal status, body weight, parity, age at menarche, age at the time of the birth of a first child and), number of past breast biopsies, and first-degree and second-degree family history of breast cancer. Women with a 5-year risk of 1.67 percent or higher are classified as "high-risk." The BCRAT has been shown to be well calibrated for predicting breast cancer risk for European American women but unfortunately not for African American women [4, 5]. Modification of the BCRAT based on data of African American women from the Women’s Contraceptive and Reproductive Experiences study also underestimated breast cancer risk in African American women [6–8].

A growing number of single-nucleotide polymorphisms (SNPs) that are associated with an increased risk of breast cancer have been identified through genome-wide association studies [9, 10]. Most of these SNPs were discovered among women of European descent. These SNPs are quite different in character from *BRCA1* and *BRCA2* mutations. While the latter confer a large increase in individual risk, they are found in very low frequencies in the population. In contrast, SNPs confer only a small increase in risk but are found in much higher frequencies in the population. While no SNP is very informative on its own, a polygenic approach to genetic screening could improve estimates of individual risk and create the possibility of individualized screening strategies [11, 12]. Several studies have shown that combining risk estimates based on SNP genotypes with risk estimates from conventional breast cancer risk prediction tools, such as the BCRAT, can improve individual risk estimates for certain ethnic populations [13–16]. Larger studies have also made efforts to identify SNPs specifically associated with breast cancer risk in African American populations through consortium including the AMBER, ROOT, and AABC [17, 18]. This approach clearly has important implications under current National Comprehensive Cancer Network guidelines for breast cancer screening and risk reduction. Such an approach has the potential to improve the efficiency of screening and chemoprevention so that more women benefit from these interventions, while at the same time reducing costs, false positives, and other harms.

The present study was initiated to confirm whether a genetic risk score based on a panel of 75 SNPs that are associated with increased breast cancer risk in women of European and/or African ancestry [19–25], could improve the BCRAT risk prediction estimates of individual breast cancer risk among a community-based cohort of African American women designed to be representative of the female population of the state of Arkansas. Our data suggests that combining a genetic risk term with a clinical risk estimate from BCRAT provides a more accurate individual risk estimate for African American women in Arkansas. These results could potentially improve targeted screening and prevention programs for African American women in Arkansas.

## Materials and methods

### Study population

Participants in this study were selected from a cohort of women recruited to study breast cancer in Arkansas [26]. Accrual began in 2007 and consists of 5,982 African American and 19,831 European American women aged 18 to 100 years who reside in Arkansas. Participants were recruited in several venues, including Susan G. Komen Race for the Cure events, American Cancer Society Relays for Life, community festivals and events, faith-based events, and community and business sponsored health fairs. A further 240 unaffected African American women were recruited at the Susan G. Komen Race for The Cure event held in April 2014 in Jackson, MS. Recruitment strategies at all events were the same and consisted of: (a) project introduction; (b) written informed consent; and (c) data collection. Details on study participant recruitment and establishment of the cohort was previously described [19].

Briefly, during recruitment to the cohort, baseline information on medical history, demographic factors, body weight, height, reproductive history, menstrual history, and family history of breast cancer, was obtained and a 2-mL saliva specimen from the participant was collected in an OrageneDNA Self Collection Kit. Self-reported race and ethnicity information was based on data obtained from questionnaires that were completed at recruitment. Participants also reported if a 1^st^ degree or 2^nd^ degree family relative (mother, sister, daughter, etc.) participated in any of the recruitment events. Participants under the age of 35 years were excluded since breast cancer risk assessment using the BCRAT algorithm is limited to women age 35 and older.

All participants in this study self-reported as African American ancestry and were selected using information in the cohort database. Study subjects included participants who had a previous diagnosis of breast cancer (n = 319), one age-matched unaffected control (n = 319) per breast cancer case, and the additional 240 unaffected controls recruited in Jackson, MS. This study and written informed consent procedure was approved by the University of Arkansas for Medical Sciences Institutional Review Board (protocol approval number 202840) and conducted in accordance with the Declaration of Helsinki.

### SNP selection

The SNP panel was based upon SNPs identified as being associated with breast cancer risk by large-scale genome-wide association studies and had previously been used to derive a validated polygenic risk score using a Caucasian data set [27]. Because many of the SNPs have subsequently been found to have odds ratios of a different magnitude, or are directionally different, for African Americans, the polygenic risk score used in this study was based on the previously reported odds ratios for African Americans [18, 21]. The 75-breast cancer associated SNPs analysed in this study, together with the odds ratios calculated for the study population and the published values for both African Americans and Caucasians, are listed in S1 Table.

### SNP genotyping

Genomic DNA was isolated from saliva specimens of the 319 cases and 559 controls using DNA Genotek’s prepIt·L2P DNA Extraction Kit (Ontario, Canada) according to the manufacturer’s instructions. Genomic DNA was evaluated and quantified using a Nanodrop UV-spectrometer (Thermo Fisher Scientific, Wilmington, DE).

Whole-genome amplification was conducted in the UAMS Genomics Core Facility using Qiagen RePLI-g Kits, which incorporates the method of multiple displacement amplification with input of 50 nanograms of genomic DNA per reaction. Amplified samples were purified and quantified on a Nanodrop 2000 (Wilmington, DE) before being plated for genotyping.

Genotyping was performed using the Illumina GoldenGate Genotyping Assay at the Winthrop P. Rockefeller Cancer Institute’s Genomics Core Facility. The GoldenGate Genotyping Assay is a flexible pre-optimized assay that uses a discriminatory DNA polymerase and ligase to interrogate 96, or from 384 to 3,072, SNP loci simultaneously. Blinded duplicate samples (5–10%) were included to assess the reproducibility of the genotypes. An average reproducibility of 99% was obtained. Individual level genotypes are presented in S2 Table.

### Risk prediction

The 5-year and lifetime absolute risk of invasive breast cancer at baseline was calculated using BCRAT [6, 28]. We excluded the personal breast cancer diagnosis for cases in the risk calculations. We did not have information on biopsies or atypical hyperplasia, so these variables were coded as ‘unknown’, which is an accepted standard response option for BCRAT. In accordance with BCRAT’s design, its application was restricted to women aged 35 years or older [6, 28]. We incorporated the modified Women’s Contraceptive and Reproductive Experiences (WeCARE) model for breast cancer risk estimation in African American women.

### SNP-based risk score and combined risk score

We used the approach of Mealiffe et al. [13] to calculate a SNP-based (relative) risk score. We determined the SNP-based risk score using estimates of the odds ratios (OR) per allele and the risk allele frequencies (*p*) from a previously published study [27], assuming independent and additive risks on the log OR scale. For each SNP, we calculated the unscaled population average risk as μ = (1 –*p*)^2^ + 2*p* (1 –*p*) OR + *p*^2^OR^2^ as previously described [25]. After the adjusted risk values were calculated [25], we calculated the overall SNP-based risk score by multiplying the adjusted risk values for each of the 75 SNPs investigated [13]. Lastly, the combined risk score was calculated by multiplying the SNP-based score by the model’s predicted 5-year/lifetime risk of breast cancer as described by Dite et al [25].

### Statistical analysis

We log transformed the BCRAT risk score, SNP-based score, and combined risk score for all analyses. To test for associations between the BCRAT risk score, the SNP-based risk score, and the combined risk score, we used Pearson correlation.

We used logistic regression to estimate risk associations for the BCRAT, SNP-based, and combined risk scores in terms of the OR per adjusted standard deviation (OPERA), where the log 5-year predicted risk was adjusted for age [29]. We assessed model calibration using the Hosmer–Lemeshow goodness-of-fit test And measured discrimination between cases and controls was measured using the area under the receiver operating characteristic (AUC) curves of the risk scores as previously described [25]. We categorized 5-year absolute risks as low risk (<1.66%) and high risk (≥1.66%) and constructed reclassification tables as a cross-tabulation of the classification of the BCRAT risk score with the classification of the combined risk score. Similarly, we categorized lifetime absolute risks as low risk (<20%) and high risk (≥20%) and constructed reclassification tables as a cross-tabulation of the classification of the BCRAT risk score with the classification of the combined risk score from. We calculated the net reclassification improvement statistic as *P*(up|case)–*P*(down|case) + *P*(down|control)–*P*(up|control) as previously described [25]. We used Stata Release 13 [30] for two sided statistical tests and all statistical analyses. A *P* value less than 0.05 was considered statistically significant.

## Results

Table 1 shows the characteristics of the study participants and the questionnaire variables used in the calculation of the BCRAT risk score. For cases, the mean age at diagnosis was 54.6 years (standard deviation [SD] = 10.4), and for controls, the mean age at interview was 53.3 years (SD = 9.8). The mean BCRAT 5-year risk of breast cancer was 1.80% (SD 1.10%) for cases and 1.20% (SD 0.50%) for controls. The mean BCRAT lifetime risk of breast cancer was 10.4% (SD 5.20%) for cases and 8.0% (SD 2.70%) for controls. The mean SNP-based risk score was 1.02 (SD 0.43) for cases and 0.89 (SD 0.38) for controls. S1 Table shows, for each of the 75 SNPS, the per-allele OR and 95% CI in this study as well as the previously published per-allele OR and 95% CI.

**Table 1:** Characteristics of study participants.

	Cases (n = 319)		Controls (n = 559)	
	N	(%)	N	(%)
Age (years)				
35–39	23	(7.2)	39	(7)
40–44	34	(10.7)	69	(12.3)
45–49	48	(15.1)	88	(15.7)
50–54	61	(19.1)	127	(22.7)
55–59	49	(15.4)	101	(18.1)
60–64	48	(15.1)	53	(9.5)
65–69	29	(9.1)	46	(8.2)
70–74	15	(4.7)	21	(3.8)
≥70	12	(3.8)	15	(2.7)
Number of first-degree relatives with breast cancer				
0	177	(55.5)	336	(60.1)
1	45	(14.1)	55	(9.8)
≥2	49	(15.4)	5	(0.9)
Missing	48	(15.1)	163	(29.2)
Age at menarche (years)				
≤9	6	(1.9)	25	(4.5)
10	30	(9.4)	53	(9.5)
11	34	(10.7)	55	(9.8)
12	94	(29.5)	166	(29.7)
13	84	(26.3)	118	(21.1)
14	23	(7.2)	55	(9.8)
15	12	(3.8)	32	(5.7)
16	16	(5)	26	(4.7)
≥17	7	(2.2)	15	(2.7)
Missing	13	(4.1)	14	(2.5)
Age at first live birth (years)				
≤19	106	(33.2)	181	(32.4)
20–24	105	(32.9)	182	(32.6)
25–29	40	(12.5)	66	(11.8)
30–34	14	(4.4)	37	(6.6)
≥35	6	(1.9)	8	(1.4)
Missing	48	(15.1)	85	(15.2)
Number of breast biopsies				
0	0	0	31	(5.6)
1	12	(3.8)	7	(1.3)
2	5	(1.6)	2	(0.4)
Missing	302	(94.7)	519	(92.8)
Had hyperplasia				
No	7	(2.2)	37	(6.6)
Yes	12	(3.8)	4	(0.7)
Missing	300	(94)	518	(92.7)

**Fig 1:**
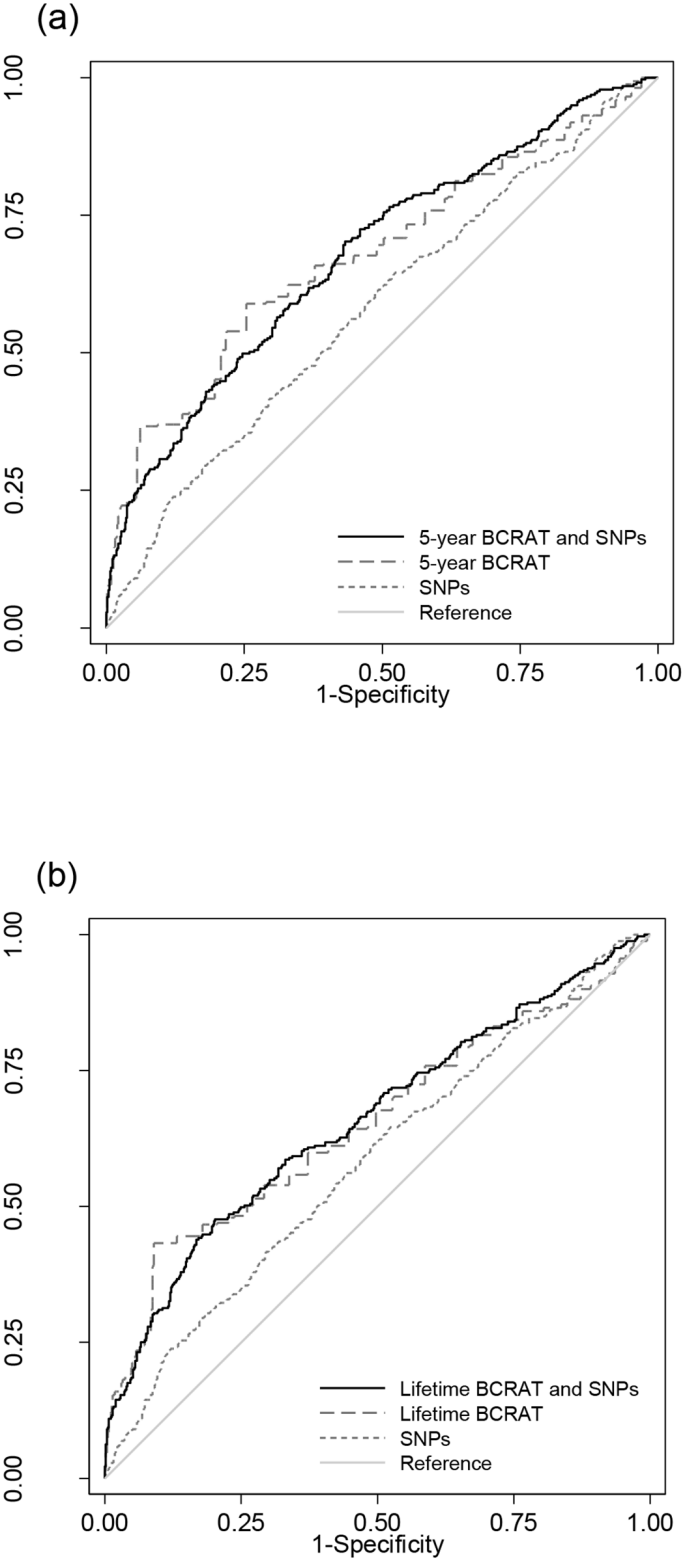
The receiver operating characteristic curves (ROC) and area under the curve (AUC) for a) 5-year BCRAT risk, single-nucleotide polymorphism (SNP) risk, and combined 5-year BCRAT × SNP risk and b) lifetime risk.

The cases in the study population were oversampled for family history; in particular 49 (15.4%) of cases had 2 or more first-degree relatives, well in excess of that observed in population-based case samples [31], This resulted in an AUC value for BCRAT which was substantially higher than previously published values, a consequence of number of affected relatives being included in the BCRAT algorithm. For the 5-year risk estimates, the combined risk score did not improve the AUC over BCRAT alone (p = 0.4), similarly for lifetime risk, the combined score did not improve the AUC (p = 0.7; see Table 2 and Fig 1).

**Table 2:** AUC for the unadjusted risk scores.

Log-transformed risk score	AUC	(95% CI)
5-year BCRAT	0.678	(0.639, 0.716)
Lifetime BCRAT	0.652	(0.612, 0.692)
SNP	0.581	(0.541, 0.620)
5-year BCRAT × SNP	0.679	(0.642, 0.716)
Lifetime BCRAT × SNP	0.658	(0.619, 0.696)

Change in AUC (1 degree of freedom).

5-year BCRAT: χ^2^ = 0.0, *P* = 1.0.

Lifetime BCRAT: χ^2^ = 0.13, *P* = 0.7.

*P*< 0.05 is considered statistically significant.

The OPERA estimates of the OR, however, did confirm that the combined risk scores for 5-year and lifetime risk gave greater discrimination than the SNP-based risk score and the corresponding BCRAT 5-year and lifetime risk scores alone (Table 3). Furthermore, incorporation of the SNP-based risk score improved the OPERA estimate from 1.84 to 2.08 for the BCRAT 5-year risk estimate and from 1.79 to 2.07 for the BCRAT lifetime risk estimate.

**Table 3:** Age-adjusted OPERA estimates of the OR, with corresponding 95% Cis.

Log-transformed risk score	OPERA OR	(95% CI)	P
5-year BCRAT	1.835	(1.611, 2.090)	< 0.001
Lifetime BCRAT	1.798	(1.589, 2.035)	< 0.001
SNP	1.434	(1.220, 1.687)	< 0.001
5-year BCRAT × SNP	2.085	(1.774, 2.449)	< 0.001
Lifetime BCRAT × SNP	2.069	(1.757, 2.438)	< 0.001

*P*< 0.05 is considered statistically significant.

Reclassification tables were used to calculate the number of cases and controls whose risk classification changed with respect to the American Society of Clinical Oncology’s 5-year risk threshold of 1.66% for Tamoxifen chemoprevention and the American Cancer Society’s lifetime risk threshold of 20% for MRI screening is recommended (Tables 4 and 5). Reclassification analysis showed that after incorporating the SNP-based risk score, 13% of cases had their 5-year risk increased above the 1.66% threshold (NRI = 0.020 (95% CI -0.040, 0.080)) and 6.3% of cases had their lifetime risk increased above the 20% threshold (NRI = 0.034 (95% CI 0.000, 0.070)).

**Table 4:** Five-year BCRAT reclassification table.

5-year BCRAT	Combined risk score	
	<1.66%	≥1.66%
Cases		
<1.66%	147	42
≥1.66%	34	96
Controls		
<1.66%	411	47
≥1.66%	44	57

NRI = 0.020 (95% CI -0.040, 0.080).

Abbreviation: NRI, net reclassification improvement.

**Table 5:** Lifetime BCRAT reclassification table.

Lifetime BCRAT	Combined risk score	
	<20%	≥20%
Cases		
<20%	279	20
≥20%	8	12
Controls		
<20%	554	3
≥20%	1	1

NRI = 0.034 (95% CI 0.000, 0.070).

Abbreviation: NRI, net reclassification improvement.

## Discussion

Genome-wide association studies conducted mainly in European or Asian populations have identified an increasing number of genetic susceptibility loci for breast cancer. In addition to the observation that SNPs are associated with increased risk of developing breast cancer, several studies have investigated the possibility of combining the SNP-based risk scores with conventional breast cancer risk prediction tools, particularly for women of European descent [13–16, 32] but also recently for women of African ancestry and Hispanic ancestry [33]. Using the same SNPs validated in a recent comprehensive panel for African Americans [25], this study’s smaller, independent cohort of African American women in Arkansas confirms the added value of SNP to breast cancer risk assessment among the African American population. Importantly, for European ancestry women, the combined SNP-based and risk prediction tool scores are now among the strongest known measures for differentiating women with and without breast cancer [32]. Given the disparity in stage at breast cancer diagnosis between African American women and other US populations, identification and testing the validity of a breast cancer risk assessment tool that may increase screening and prevention awareness in African American women is needed and SNP-based risk assessment may enable the medical community to better identify those women that would benefit from increased screening, screening adherence or risk reducing medication.

We have evaluated the effect of combining a polygenic risk score with the BCRAT risk score (incorporating the modified Women’s Contraceptive and Reproductive Experiences WeCARE model) for breast cancer risk estimation in African American women. SNP-based risk has been shown to be largely independent of family history with only a modest attenuation when adjusting for family history [27]. In this study, the AUC did not significantly improve for the 5-year or lifetime risk estimates by the addition of the SNP-based risk score. Receiver operating characteristic curve analysis has previously been criticized in the context of risk prediction test performance [13, 34], and it is our view that the AUC values obtained for the BCRAT in this study were inflated due to the enriched and strong family history of the cases resulting in an AUC substantially higher than that previously reported for African American women unselected for family history [33].

We believe a better way to combine risk factors when they are measured using different scales is to utilize methodologies in which the standard deviation of the risk gradient is incorporated into the analysis. We have therefore, preferentially utilized the odds per adjusted standard deviation method (OPERA), as described by Hopper 2016 [29]. The concept of OPERA allows comparison of the ability of risk factors to differentiate cases from controls on a population, as distinct from individual, basis and can be applied to continuous, binary, and ordinal risk factors. Following the OPERA concept, we have presented the OR in terms of the age-adjusted standard deviation of the risk score. OPERA is based on the fact that the estimated risk gradient for a risk factor is its change when holding constant all other factors that have been controlled for by design or analysis. That is why we fitted the age-adjusted risk scores. If the OR is the risk gradient on a given scale, then OPERA = OR^*s*^, where *s* is the estimated SD of the risk factor in the population after adjusting for all other factors (and can be estimated using the controls). Under these terms of reference, the addition of the SNP-based risk score resulted in a 13% improvement in the OPERA for the BCRAT 5-year risk estimate and a 15% improvement for the BCRAT lifetime risk estimate. This improvement in risk assessment might help the medical community begin to tackle the differences in stage at diagnosis; significantly fewer Non-Hispanic blacks are diagnosed at a localized stage compared to non-Hispanic whites [35].

We acknowledge that a limitation of the present study included small participant sample size and lack of data pathological subtypes of breast cancer among the participants. Within the cohort of women used for this study population, many were also unable to complete the BCRAT in its entirety (Table 1). However, because the outcome of these data focuses on the change as a result of the incorporation of SNP-based risk, the baseline missing BCRAT data would not significantly alter the combined effects of SNP-based change. However, as identified above, the cases are enriched for family history, which has impacted the BCRAT test performance.

Reclassification of individual risk is important if the reclassification results in an individual’s risk exceeding clinical guideline thresholds, such as those for chemoprevention or screening. Reclassification analysis showed that 22% of cases that were previously below the American Society of Clinical Oncology’s risk threshold of 1.66% for Tamoxifen chemoprevention would have their individual risk elevated above the threshold after incorporation of the SNP-based risk score. Similarly, 6.7% of cases who were previously below the 20% lifetime risk threshold for MRI screening would have their risk elevated above the threshold by the addition of the SNP-based risk score.

The unique feature of our population-based cohort is that the women are representative of 75 counties, encompassing both rural and urban areas, within the state of Arkansas [19]. Arkansas has one of the highest breast cancer mortality rates among women of color [1] and; therefore, our results could potentially improve targeted screening and prevention programs for African American women in Arkansas.

## Conclusions

In conclusion, our results indicate that discriminatory accuracy of BCRAT is improved for African American women in Arkansas with the inclusion of African American specific SNP breast cancer risk alleles. We anticipate that the SNP-based risk score will further improve when genome-wide association studies use Phase I data sets that are relevant to the African American population, and when fine mapping studies have been conducted within that population.

## Supporting information

S1 Table Unadjusted per-allele ORs for individual SNPs and the previously published ORs used to derive the risk scores. (DOCX)

S2 Table Individual level genotypes of participants in our study. (CSV)
